# 976. Latent Profile Analysis of PROMIS-29 Domains in Burn Survivors: Analysis and Comparison to General Population

**DOI:** 10.1093/jbcr/irag033.573

**Published:** 2026-04-06

**Authors:** Andrew Humbert, Kara McMullen, Bill Insko, Karen J Kowalske, Fenan Rassu, Jeffrey C Schneider, Sarah A Stoycos, Dagmar Amtmann

**Affiliations:** Department of Rehabilitation Medicine, University of Washington, Seattle, WA; Department of Rehabilitation Medicine, University of Washington, Seattle, WA; Department of Rehabilitation Medicine, University of Washington, Seattle, WA; Department of Physical Medicine and Rehabilitation, University of Texas Southwest Medical Center, Dallas, TX; Johns Hopkins University School of Medicine, Baltimore, MD; Spaulding Rehabilitation Hospital, Mass General Brigham, Harvard Medical School, Boston, MA; Keck School of Medicine, University of Southern California, Los Angeles, CA; University of Washington, Seattle, WA

## Abstract

**Introduction:**

While burn injuries impact physical and mental health as measured by self-reported health outcomes, no known work has identified outcome profiles across multiple physical and mental health domains. This work aims to identify unique classes of outcome profiles in people with burn injuries, compare these classes to those previously identified in adults with chronic conditions, and compare demographic and injury characteristics between classes.

**Methods:**

Utilizing data from a large, national burn outcome database, a latent profile analysis was conducted to identify unique class profiles at 6 months following discharge across all PROMIS 29 domains, which consists of physical function, anxiety, depression, fatigue, sleep disturbance, ability to participate in social roles and activities, pain interference, and pain intensity. A variety of fit metrics (Bayesian information criterion, entropy, and class size) were used to determine the optimal number of classes. Summary statistics were used to compare patient reported outcomes, demographic, and injury characteristics across classes from the optimum model.

**Results:**

A total of 894 participants had complete PROMIS-29 data available at 6-months following discharge and were included in the analysis. Although no single model was uniformly superior, a 5-class model was determined most appropriate. The 5 classes correspond to those with very severe outcomes (n = 72), severe outcomes (n = 235), moderate outcomes with worse mental health outcomes(n = 112), moderate outcomes with worse pain (n = 188), and minimally impacted outcomes (n = 287).

**Conclusions:**

Participants’ scores were generally consistent across PROMIS-29 domains, with severe scores in one domain indicating severe scores across all domains. These classes approximately corresponded to previously identified classes in a general population of adults with chronic conditions. While differences in demographic and injury characteristics between classes were apparent, no uniform trend was present across the severity classes this work identified.

**Applicability of Research to Practice:**

These findings indicate those with poor outcomes in any one domain likely need support across a wide range of physical and mental health domains.

**Funding for the study:**

The contents of this manuscript were developed under grants from the National Institute on Disability, Independent Living, and Rehabilitation Research.

